# An easy and novel method for safer brachytherapy: real-time fluoroscopic verification of high-dose-rate ^192^Ir source position using a flat-panel detector

**DOI:** 10.1093/jrr/rrz013

**Published:** 2019-04-24

**Authors:** Takayuki Nose, Koji Masui, Tadashi Takenaka, Hideya Yamazaki, Katsuya Nakata, Yuki Otani, Shinichiro Kumita

**Affiliations:** 1 Department of Radiation Oncology, Nippon Medical School, Tamanagayama Hospital, Nagayama, Tama, Tokyo, Japan; 2 Department of Radiation Oncology, University Hospital, Kyoto Prefectural University of Medicine, Kajii-cho,Kawaramachi-Hirokoji, Kamigyo, Kyoto, Kyoto, Japan; 3 Department of Radiology, Kaizuka City Hospital, Hori, Kaizuka, Osaka, Japan; 4 Department of Radiology, Nippon Medical School Hospital, Sendagi,Bunkyo, Tokyo, Japan

**Keywords:** high-dose-rate, brachytherapy, real-time, verification, flat-panel detector, fluoroscopy

## Abstract

Real-time fluoroscopic verification of the active source position during actual treatment is the only established method to prevent high-dose-rate (HDR) brachytherapy events. The challenge is spurious signals from an HDR ^192^Ir source that result in image halation, making source positions indiscernible when using a non-modified image intensifier fluoroscope. We have previously reported a method for observing an HDR ^192^Ir source by using an elaborately modified image intensifier system. The newly developed flat-panel detector fluoroscope is, by contrast, inherently halation-free thanks to the wider dynamic range (12–14 bits), compared with image intensifier fluoroscopes (8 bits). To explore the feasibility, we applied a commercially available flat-panel detector fluoroscope without modification to actual treatment. We successfully observed source positions without halation for all 107 patients, with a total of 522 HDR treatment sessions during a 3-year period from 2014 to 2017. Actual source positions were compared with planned positions on the planning hard copy. With this method, we detected a total of 1 error (0.2%) among the 522 sessions, at a similar detection rate of 0.1% with our previous experience using a modified image intensifier fluoroscope. We found that a commercially available flat-panel detector fluoroscope is ready for use for real-time verification and outweighs the need for elaborate modifications of an image intensifier fluoroscope. A flat-panel detector fluoroscope will help the global radiation oncology community promote real-time verification programs, leading to safer HDR brachytherapy.

## INTRODUCTION

Although rare, events that led to one patient death elsewhere have been reported for high-dose-rate (HDR) brachytherapy treatments [1]. Real-time fluoroscopic verification of HDR ^192^Ir source position during treatment using a modified image intensifier (II) system has been the only practically established method for preventing accidents [2]. To observe an HDR ^192^Ir source, an II system, however, needs dedicated, elaborate modifications to avoid halation due to scattered photons from the HDR ^192^Ir source itself [2].

In contrast to II systems (dynamic range, 8 bits), newly developed flat-panel detector (FPD) systems are, thanks to the wider dynamic range (12–14 bits), practically halation-free, with finer gradation processing [3, 4]. At Shimane University Hospital, an FPD system was, for the first time, applied to a routine quality control program to check dwell position accuracy for the HDR ^192^Ir source. Successful, clear X-ray images resulted from effective use of the FPD system, in which a minimum dislocation of 0.04 mm was detectable [3, 4].

Inspired by these reports, we decided to explore the applicability of the FPD system to real-time fluoroscopic verification of an HDR ^192^Ir source position during actual treatment.

## MATERIALS AND METHODS

**Fig. 1.**
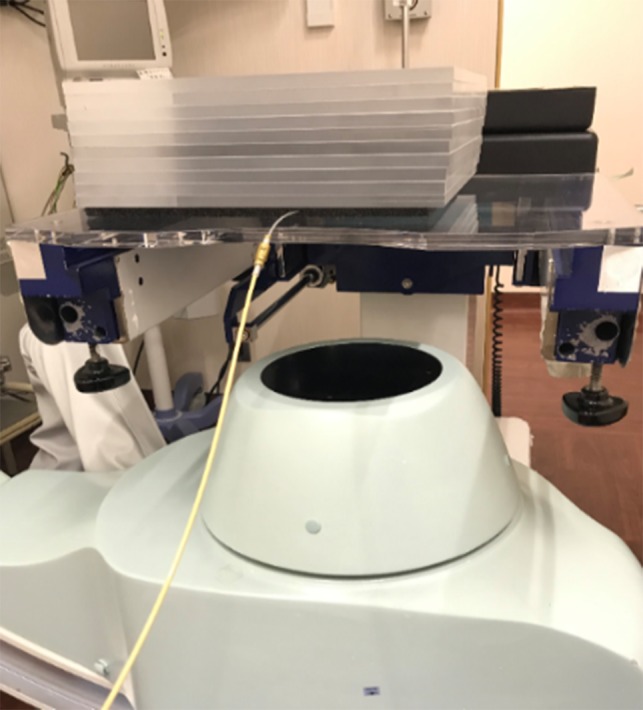
Commissioning test set-up: an X-ray test chart (not shown) mounted on a 100 mm-thick phantom (300 mm × 300 mm × 100 mm), an HDR applicator connected to a joint tube set beneath the phantom, and an X-ray tube for the flat-panel detector system positioned under the couch.

**Fig. 2.**
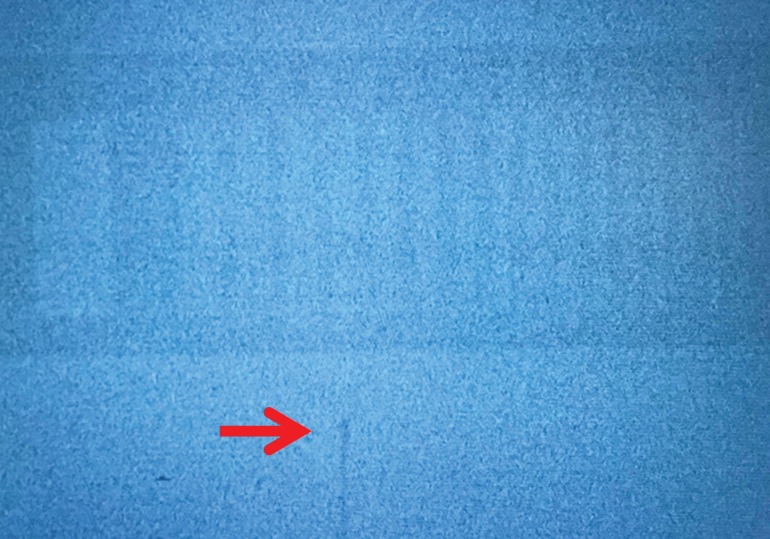
Fluoroscopic monitor image of an X-ray test chart and an HDR ^192^Ir source with the automatic exposure control function turned on. Image quality was not clear enough to observe the test chart and the ^192^Ir source (arrow).

At the Hospital of Kyoto Prefectural University of Medicine, an FPD C-arm system (Safire®, Shimadzu, Kyoto, Japan) was installed in 2014. We then conducted a commissioning test for our clinical objective of the HDR ^192^Ir source fluoroscopy by using an X-ray test chart Type 2 (1.0–4.86 LP/mm, Moriyama X-Ray Equipments Co. Ltd, Tokyo, Japan) mounted on a 100 mm thick acrylic phantom (Fig. 1). We found that the automatic exposure control function interfered with image quality in the presence of an HDR ^192^Ir (Fig. 2), so we routinely turned off this function during source fluoroscopy. For this test, X-ray with 80 kV and 6.6 mA was used, and the source–image distance was set at 1200 mm. We verified the visibility of 0.5 mm-thick lines for 1.0 LP/mm in the presence and absence of an HDR ^192^Ir source set beneath the phantom. This test has also been performed as a yearly check thereafter. After confirming clear visibility of 0.5 mm-thick chart lines and an HDR ^192^Ir source (0.9 mm × 4.5 mm) in the commissioning test, we started to apply this verification method for all actual treatment sessions using microSelectron HDR-v3 (Nucletron, Elekta AB, Stockholm, Sweden). We verified actual dwell positions relative to planned positions in a planning hard copy, as described elsewhere [2]. For a reference hard copy preparation, all the dwell positions, which were viewed approximately from the planned fluoroscopic angle, were printed out beforehand. To collimate the axes of the observer’s view and the reference hard copy’s view, the technician adjusted the C-arm position to focus on the implant from the planned angle before starting every treatment session. The responsible radiation oncologist(s) and an expert technician together observed the fluoroscope monitor, the reference hard copy, and the HDR console monitor. When at least one observer suspected a dislocation with no less than an active source length of 4.5 mm, the technician, without discussion or agreement, immediately canceled the treatment by pushing the interruption button. Then, the treatment parameters, including the length and connection of the joint tubes, were double-checked by at least two staff members. If an error was confirmed, the treatment was resumed only after the appropriate correction was made.

To lower the fluoroscopic exposure, we tried to shorten the fluoroscopic time by utilizing an instantaneously captured fluoroscopic image for each dwell position instead of using continuous fluoroscopy. We also reduced the fluoroscopic pulse rate and the frame rate to a quarter of the standard 30 frames per second to 7.5 frames per second. At this rate, the movement of an ^192^Ir source was still sufficiently smooth to track, as shown in our previous study and the attached videos [2] (https://www.redjournal.org/article/S0360-3016(16)33543-X/addons, last accessed April 7, 2019).

Before the commencement of the study, the protocol was approved by the institutional review board. Patients were informed of the study purposes, and written informed consent was obtained before participation. During the 3-year period from 2014 to 2017, we treated 107 patients by HDR brachytherapy, with a total of 522 treatment sessions, all of which (100%) were monitored using the current FPD C-arm fluoroscope.

## RESULTS

**Fig. 3.**
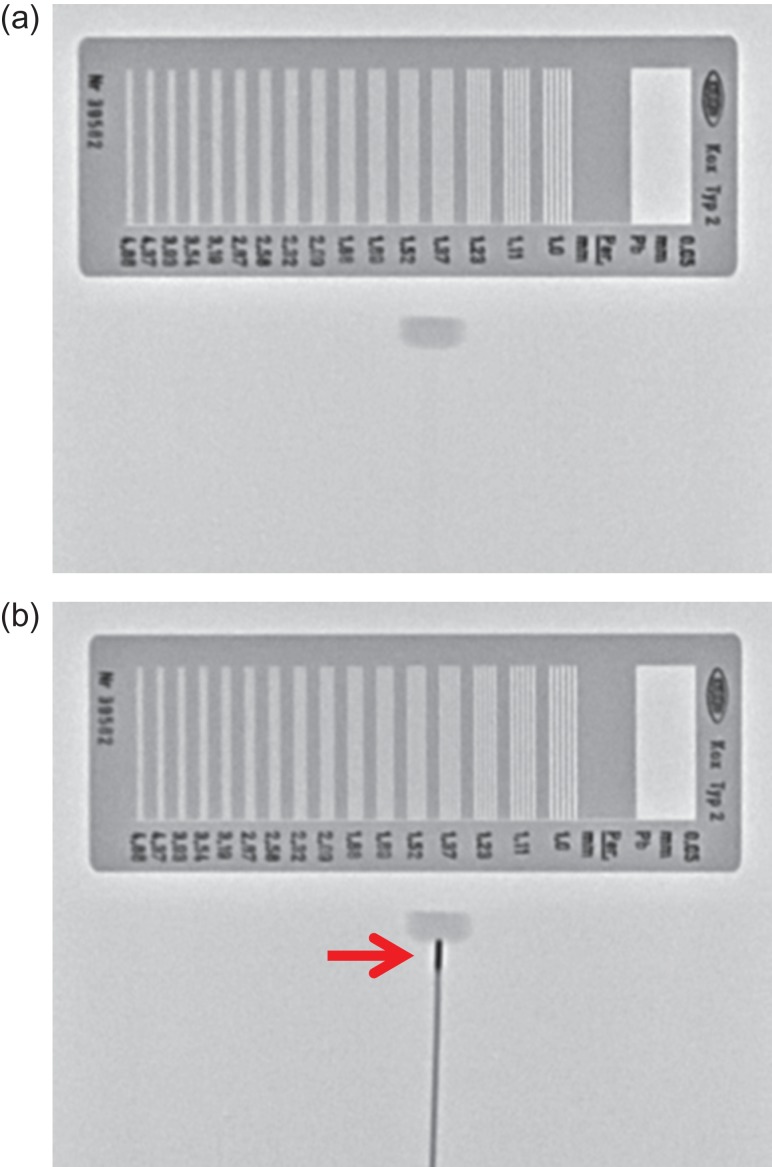
(a) Fluoroscopic image of an X-ray test chart mounted on a phantom. (b) Fluoroscopic image of an X-ray test chart mounted on a phantom and an active HDR ^192^Ir source (arrow) beneath the phantom.

For the commissioning test, the 0.5 mm-thick chart lines were confirmed to be visibly resolved, either with or without an HDR ^192^Ir source (Fig. 3a and b). The HDR ^192^Ir source (0.9 mm × 4.5 mm) was also visible, as shown in Fig. 3b.

**Fig. 4.**
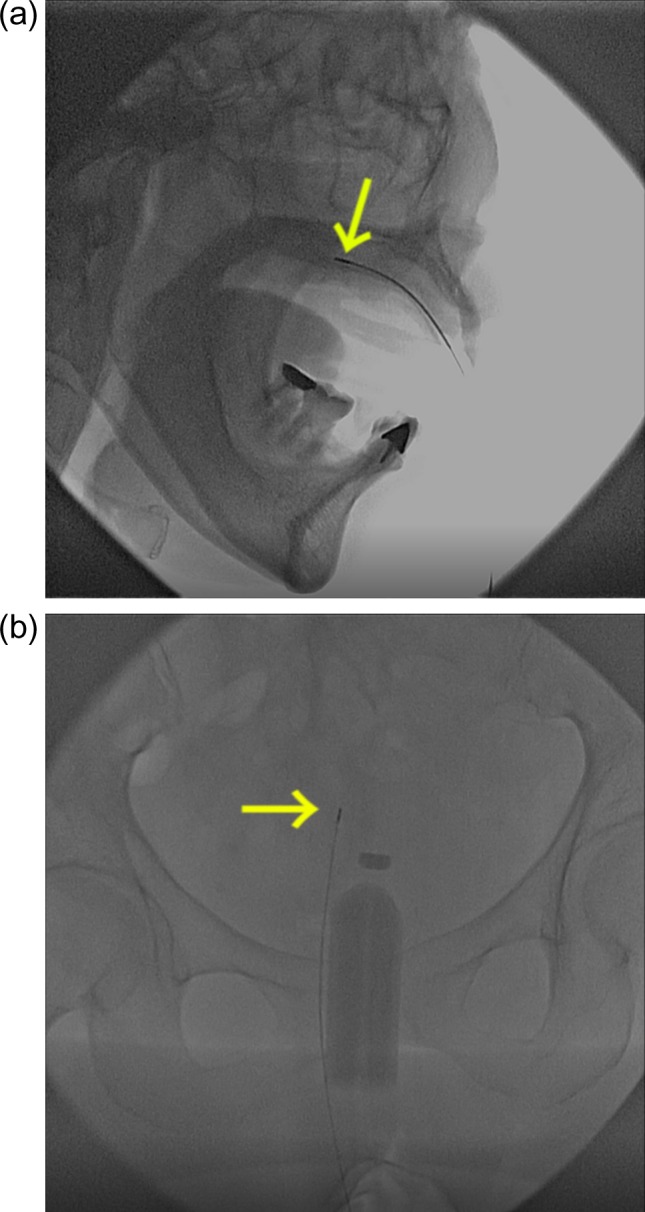
(a) A patient with cancer of the hard palate treated with mold brachytherapy. (b) A patient with gynecologic cancer treated with interstitial brachytherapy. Arrows indicate the active source at the top of the drive cable.

The current FPD system also acquired clear images for actual HDR brachytherapy. Examples of brachytherapy for hard palate and gynecologic cancers are presented in Fig. 4. As depicted in Fig 3b, 4a and 4b, this fluoroscopic system provided excellent image quality with which we could observe an actual source of 0.9 mm × 4.5 mm clearly relative to the anatomy.

With this method, we detected a total of 1 error (0.2%) among the 522 treatment sessions performed during the 3 years. This error was detected in a patient with gingival osteosarcoma treated with interstitial brachytherapy. For this patient, both observers found the ^192^Ir source did not dwell at the planned first dwell position, with a dislocation apparently greater than the source length, compared with the planning hard copy. We promptly stopped treatment at the wrong first position. On a subsequent double-check, we found that two applicators had been connected by inadvertently switched joint tubes. After error correction, we successfully executed the correct treatment. For the remaining 521 sessions, we observed no dislocation with no less than an active source length of 4.5 mm.

## DISCUSSION

To the best of our knowledge, the current study is the first to confirm the applicability of an FPD system to real-time fluoroscopic verification of HDR ^192^Ir source positions during treatment.

Using an II system without dedicated modifications, we could not achieve fluoroscopic verification of HDR ^192^Ir source positions [2]. Photons from the HDR ^192^Ir source interacted with the patient and generated scattered photons containing only spurious signals and quantum noise. These scattered photons interacted with the II system and markedly degraded X-ray image quality by lowering the image contrast to <20% and the signal-to-noise ratio to <60% [5]. The degraded images resulted only in halation because of the insufficient dynamic range (8-bit) of the II system. Neither anatomical information nor the ^192^Ir source locations were discernible. To avoid halation and to obtain sufficient image quality with an II system, we made modifications by increasing the following values by a factor of 4: X-ray dose per pulse; X-ray pulse interval; and frames per second. This increased X-ray information, avoided overexposure, and projected only non-degraded images on the monitor. With these elaborate modifications, we could finally execute fluoroscopic verification of an HDR ^192^Ir source position [2].

By contrast, using a commercially available FPD system, we could simply execute fluoroscopic verification of an HDR ^192^Ir source position without any modifications, since FPD systems are practically halation-free thanks to the wider dynamic range (12- to 14-bit).

Our previous study reviewed the literature on HDR treatment events; 80% of events were deemed detectable by fluoroscopic verification. Actually, we detected 2 treatment errors (0.1%) among 2034 treatment sessions using the modified II system [2]. In the current study using the FPD system, we detected errors at a similar detection rate of 0.2%.

Fluoroscopic verification is, for the moment, the only practically validated real-time verification method for HDR brachytherapy treatment. The necessity of elaborate modifications for II systems, however, may disincline brachytherapy departments to install II systems. Moreover, most manufacturers have shifted to production of FPD systems over II systems. FPD systems that need no modifications are, therefore, the more practical and realistic option for real-time verification. We believe installing an FPD system will help the global radiation oncology community promote real-time verification programs, leading to safer HDR brachytherapy.
